# The Association between Physical Activity and CAMDEX-DS Changes Prior to the Onset of Alzheimer’s Disease in Down Syndrome

**DOI:** 10.3390/jcm10091882

**Published:** 2021-04-27

**Authors:** Sarah E. Pape, R. Asaad Baksh, Carla Startin, Sarah Hamburg, Rosalyn Hithersay, Andre Strydom

**Affiliations:** 1 Institute of Psychiatry, Psychology, and Neuroscience, King’s College London, London SE5 8AF, UK; Carla.Startin@roehampton.ac.uk (C.S.); rosalyn.hithersay@kcl.ac.uk (R.H.); andre.strydom@kcl.ac.uk (A.S.); 2 Behavioural and Developmental Psychiatry Clinical Academic Group, South London and Maudsley NHS Foundation Trust, London BR3 3BX, UK; 3 Department of Psychology, University of Roehampton, London SW15 5PJ, UK; 4 Division of Psychiatry, University College London, London W1T 7NF, UK; 5 The LonDowns Consortium, Institute of Psychiatry, Psychology, and Neuroscience, King’s College London, London SE5 8AF, UK; s.hamburg@alumni.ucl.ac.uk

**Keywords:** dementia, Down syndrome, exercise, modifiable risk factors, cognitive decline, Alzheimer’s disease, physical activity

## Abstract

Background: People with Down syndrome are at ultra-high risk of developing Alzheimer’s dementia. At present, there are no preventative or curative treatments. Evidence from sporadic Alzheimer’s disease literature suggests that lifestyle factors including physical activity may help maintain cognitive and functional skills and reduce dementia risk. Our study aimed to explore the association between regular exercise undertaken by participants with Down syndrome and changes in dementia-related domains of cognition and function. This was to consider whether physical activity may be a protective measure to delay cognitive decline and dementia in Down syndrome. Methods: Demographic, lifestyle, and health information was collected at baseline and at a two year follow up from 214 adults with Down syndrome without dementia, who also underwent assessment using the Cambridge Examination for Mental Disorders of Older People with Down Syndrome and Others with Intellectual Disabilities (CAMDEX-DS) and genetic analysis. Logistic regression models were used to examine the potential associations between decline in CAMDEX-DS domains and exercise whilst controlling for key variables. Results: At baseline, engaging in moderate intensity exercise was associated with a 47% reduced risk of everyday skills decline and engaging in high intensity exercise was associated with a 62% reduced risk of decline in personality and behaviour. At follow-up, high levels of exercise were associated with an 87% reduced risk of decline in personality and behaviour. Moderate intensity exercise at baseline was associated with a 62% reduction in risk of decline during the follow-up period in memory and orientation. Discussion: Based on our data it appears that regular moderate and high intensity exercise could reduce the risk of clinically detectable decline in a Down syndrome population with possible long-term benefits. People with Down syndrome may engage in less physical activity than their peers, and barriers remain which can prevent people with Down syndrome engaging in exercise. Our work highlights how important it is that people with Down syndrome are supported to be physically active, and to promote exercise as part of a healthy ageing plan. Clinical trials in this area would be justified to determine if engaging in exercise can lead to realistic improvements in maintaining functioning and delaying dementia onset in Down syndrome and to help develop guidance in this area.

## 1. Introduction

Down syndrome (DS) is a chromosomal disorder most commonly caused by triplication of chromosome 21. It affects around 1 in 800 to 1 in 1000 live births globally and is the most common genetic cause of intellectual disability (ID) [1,2,3]. As life expectancy of adults with DS has dramatically improved, it has become evident that they are also at ultra-high risk of developing AD at an earlier age than the general population [4,5,6]. Characteristic neuropathologic changes are observed with amyloid-beta (Aβ) plaques and neurofibrillary tangles present in brain tissue [7,8,9]. 

The primary cause of the high AD risk is triplication of the amyloid precursor protein (APP) gene located on chromosome 21 [10]. However, despite this common genetic driver there is considerable variation in the age of symptom onset, speed of progression, and pattern of decline [11]. Some of these variations have been attributed to other genetic factors such as the presence of APOε4 alleles, which are associated with increased risk of AD in the general population [12] and an earlier age of AD onset in DS [13].

Modifiable or “lifestyle” factors have also been suggested as possible modulators of dementia risk. Studies in sporadic onset AD (SOAD) have suggested associations between factors such as obesity, smoking, poor diet, high blood pressure, cardiovascular disease (CVD), fewer years in education, low levels of socialisation, reduced exercise, raised cholesterol and alcohol consumption with worse cognitive health and increased risk of dementia [14,15]. A population-based analysis concluded that relative reductions of 10% per decade in the prevalence of seven such risk factors could reduce the worldwide prevalence of AD by 8.3% by the year 2050, with approximately one third of SOAD cases worldwide potentially attributable to modifiable risk factors [16]. These factors also appear to be relevant to those with genetic drivers of AD [17].

Reports in the literature indicate potential neuroprotective effects of physical activity (PA) and an inverse relationship between PA and dementia risk in the general population [18,19]. The association between levels of PA and cognitive decline in Down syndrome is argued to be of particular relevance for a number of reasons. There are disparities in the amount of PA people with Down syndrome engage in, with many not having equal opportunity to participate in regular activity [20,21,22,23]; methods to increase PA are cheap, adaptable, and easily attainable; PA can positively affect other areas of life including fitness, balance and social participation [24,25,26]; and higher levels of PA could be implemented from early life with limited ethical concerns as a form of pre-symptomatic disease modification. In addition, people with Down syndrome in the UK generally do not smoke or drink excess amounts of alcohol and have low rates of hypertension and cholesterol-related illnesses [27,28,29,30,31]. They are therefore an important population in which to study the effects of PA that are not associated with lowering general cardiovascular risk. 

Compared to the literature in SOAD, there have been relatively few studies into the effects of PA on cognition and dementia in Down syndrome. Experiments using Ts65Dn mice, a model for Down syndrome, provide initial evidence that PA could play a role in enhancing cognition. For example, voluntary wheel running in mice has been associated with better performance in cognitive tasks compared to sedentary controls, particularly when considering hippocampal mediated processes which are integral to AD progression [32,33,34]. Alongside cognitive changes, mice studies have also demonstrated effects of PA on gene and protein expression, neurogenesis, and morphology in the brain [32,33,35].

In human studies involving people with Down syndrome, episodic memory improvement has been associated with participation in a 12-week exercise programme [36]. Improvements have also been reported in post-intervention measures of executive function including inhibition [37,38], attention shifting [39], reaction time [40,41] and semantic fluency [42,43], although effect size and results vary between studies. This may be related to variations in the intensity, type and consistency of PA involved, as well as the study design. One study compared lifestyle factors including physical activity in a group of individuals with Down syndrome and AD to a sample of adults with Down syndrome but no clinical signs of AD [44]. No significant difference was observed between the groups in levels of PA. However, there was missing data in the sample, and it is noted that across the groups the levels of PA were low. 

Overall, the current literature is suggestive of a positive relationship between PA and cognitive functioning in Down syndrome, with PA having possible neuromodulating and neuroprotective effects. Further research is needed in larger samples with consistent measures to better understand the link between PA, cognition, and dementia in Down syndrome. Our study therefore aimed to explore the association between the amount of regular exercise undertaken by participants and longitudinal changes in dementia-related domains of cognition and function prior to AD onset. This was to consider whether physical activity could be a protective measure to delay cognitive decline and dementia in Down syndrome.

## 2. Materials and Methods

### 2.1. Participants

Participants were taken from the LonDowns cohort, an observational and longitudinal cohort study of individuals with Down syndrome. The cohort has been described previously [45] and consists of people over the age of 16 years with DS living in England and Wales. All adults with a clinical diagnosis of DS were eligible for inclusion. Those with acute physical or mental health conditions were excluded until recovered. 

Ethical approval for the LonDowns study was granted from the North West Wales Research Ethics Committee (13/WA/0194). Written informed consent was obtained from individuals with capacity to consent after a full explanation of the study. In the event that individuals lacked capacity to provide informed consent, a consultee signed a form on their behalf to indicate their decision regarding the individual’s inclusion based on their knowledge of the individual and his/her wishes. This is in accordance with the UK Mental Capacity Act 2005.

Participants with a clinical diagnosis of dementia at baseline (T1) or at follow up two years later (T2) were removed from the dataset. This is because we were interested in the changes in CAMDEX-DS domains prior to a dementia diagnosis and to avoid the potential confounding effect of dementia on exercise levels. Eighty-six participants had dementia at T1 (mean age 54.59, SD 6.75) and were removed from the analysis. Eleven had converted to dementia at T2 (mean age 56.8, S.D. 4.81). Our sample therefore consisted of data from 214 participants with data at T1 (122 males) and 91 participants at T2 (54 males). The smaller group at T2 is due to the loss of participants who converted to dementia, those lost to follow up, and those with incomplete data at T2. 

### 2.2. Measures

The CAMDEX-DS (Cambridge Examination for Mental Disorders of Older People with Down Syndrome and Others with Intellectual Disabilities) is an informant-based interview used to identify symptoms of decline in cognition, adaptive functioning, or behaviour, which are suggestive of early dementia-related change [46,47]. It assesses the following domains: everyday skills, memory and orientation, other cognitive skills (covering general mental functioning, language, perception, praxis, and executive functions), personality and behaviour, and self-care. Example questions include: “Does he/she have difficulty with shopping?” (everyday skills); “Does he/she have difficulty in remembering short lists of items” (memory and orientation); “Does he/she have difficulty in following instructions (other cognitive skills); “Is he she often irritable/angry” (personality and behaviour); “Does he/she have difficulty with bathing?” (self-care). For each domain a number of questions prompt the informant to state whether there has been no deterioration, a slight deterioration, or a great deterioration in that particular area. For this study a symptom-level definition of decline was used based on results from the CAMDEX-DS interview. The presence of dementia-related symptoms was classified using a binary ‘no decline’ or ‘decline’ categorisation based on informant responses for each participant. For each domain, participants were assigned to one of two groups based on whether they exhibited deterioration in their abilities compared to their best ever functioning. If one ‘great’ deterioration or two ‘slight’ deteriorations were recorded on individual CAMDEX-DS items, then the participant was regarded as showing a decline in this domain. Participants were considered to show no decline in a domain if no items were recorded as having a great deterioration, or there were less than two slight deteriorations reported. 

Participants’ current average exercise levels were split into three categories: low, moderate, and high intensity. Coding for this variable was based on informant data relating to participants’ weekly activity levels collected during a semi-structured interview. Open questions about weekly activity and exercise levels were followed-up with focused inquiries to establish the type, duration, and frequency of PA during an average week for the participant. This approach was adapted from the methods used in Kenshole et al. (2017) and Laurin et al. (2001) [44,48]. The following criteria were used for group classification:

- High intensity. Exercise of an intensity greater than walking at least three times a week, or for at least three hours a week.
- Moderate intensity. Exercise at an intensity equal to that of walking at least three times a week, or exercise less than three hours a week/three times a week of an intensity greater than walking, or at least two hours a week of exercise where intensity or type was not specified, or a combination of walking and other exercise when duration or intensity not specified.
- Low intensity. All exercise not meeting the above thresholds, including individuals who do not engage in any exercise.

Using medical history and current medication data, participants were coded as having CVD or high cholesterol. APOE genotype was determined from saliva or blood samples. As outlined in previous studies, this was performed using a Thermo Fisher Scientific Taqman assay for rs7412 and rs429358 SNPs [49,50]. Participants were classified as ε4 carriers if they had ε3:ε4 or ε4:ε4 genotype. The non-ε4 group where those without an ε4 allele (ε2:ε2, ε2:ε3 or ε3:ε3). We excluded any participants with a ε2:ε4 genotype from the dataset due to the possible counteracting effects of the ε2 and ε4 alleles [51]. 

### 2.3. Statistical Analysis

Logistic regression models were used to examine the associations between decline in CAMDEX-DS domains and PA while adjusting for the effect of age, sex (male and female), intellectual disability level (ID; mild, moderate, and severe), APOE genotype (non-ε4 carriers and ε4 carriers), CVD and high cholesterol. For T2 models we additionally included exercise levels at T1 to examine if PA at baseline was associated with potential decline at follow-up. Participants required data for at least one outcome domain on the CAMDEX-DS at T1 or T2 for inclusion. The reference groups for categorical variables were female sex, ID level: mild, non-e4 carriers, no CVD, no history of high cholesterol, low intensity of exercise. Hence, we compared those who engage in moderate or high intensity PA to those who perform very little or no PA. 

## 3. Results

Participants ages ranged between 17 and 74 years. As shown in Table 1, the mean age at T1 was 46.11 (S.D 10.47) and 49.04 (S.D. 6.32) at T2. The average follow-up time in months between visits was 24.12 (S.D = 0.81) ranging from 23 to 28 months. The age difference between participants at T1 and T2 was 2.93 years and there were approximately the same proportion of males and females in our cohort. Over 85% of our participants were white, the majority had either mild or moderate ID and were not ε4 carriers. Nearly a third of participants had CVD at T1 and this increased to 35% at T2, however very few participants had high cholesterol, were smokers, or drank alcohol at either timepoints. The majority of individuals participated in low or moderate intensity exercise.

Declines in cognition, adaptive functioning and personality and behaviour were common in our sample at both timepoints (Table 2). The most common domains of change at T1 were personality and behaviour followed by memory and orientation and other cognitive skills and then everyday skills and self-care abilities. At T2, the domains that showed the highest prevalence of decline were personality and behaviour and other cognitive skills. Approximately the same percentage of participants exhibited declines in everyday skills and memory and orientation abilities, and again decline in self-care abilities was reported in the fewest participants.

To examine the associations between CAMDEX-DS domains and PA at T1, we fitted regression models that included the domains of the CAMDEX-DS in separate models while adjusting age, sex, ID, APOE genotype and CVD. At T2, the same models were fitted with T2 data, but we also included PA at T1 to examine the longitudinal effects of exercise on potential decline at follow-up. Due to the low prevalence of high cholesterol in our sample (1.9% at T1 and 2.2% at T2) this variable was not included in the regression models.

At T1 we found that people who engaged in moderate intensity PA were significantly less likely to exhibit decline in everyday skills (RR = 0.53 (95% CI 0.26–0.95), *p* = 0.03). Specifically, for these individuals, moderate levels of PA were associated with a 47% reduced risk of experiencing changes in everyday skills compared to people who participated in low levels of PA. High intensity PA was associated with decreased risk of changes in personality and behaviour (RR = 0.38 (0.11–0.92), *p* = 0.042). Here, engaging in high intensity PA was associated with a 62% reduction in risk of decline compared to those who engaged in low levels of PA. No other CAMDEX-DS domain was associated with PA (all *p* > 0.05).

Age at T1 was associated with an increased risk of decline in memory and orientation (RR = 1.03 (1.00–1.05), *p* = 0.03) and self-care abilities (RR = 1.03, (1.01–1.06), *p* = 0.02), while severe/profound ID was associated with increased risk of decline in other cognitive skills (RR= 1.73 (1.06–2.35), *p* = 0.03). Additionally, having an APOE ε4 allele was significantly associated with increased risk of decline in memory and orientation (RR = 1.77 (1.17–2.31), *p* = 0.01). 

We found that moderate intensity PA at T1 was associated with a 62% reduced risk of decline at T2 in memory and orientation compared to low intensity PA (RR = 0.38 (0.10–0.97), *p* = 0.047). High levels of PA at T2 were associated with reduced risk of decline in personality and behaviour (RR = 0.13 (0.005–0.83), *p* = 0.043), here high levels of PA resulted in an 87% reduction in risk of decline compared to low intensity PA. 

Again, age at T2 was associated with increased risk of decline in self-care abilities (RR = 1.12 (1.04–1.21), *p* = 0.005) and we found that people with moderate ID had a 156% increase in risk of decline in personality and behaviour (RR = 2.56 (1.38–3.58), *p* = 0.008). A history of cardiovascular disease was not associated with decline at T1 or T2. 

In summary, moderate, and high levels of exercise may be protective factors against cognitive and functional decline in adults with DS. A reduced risk of decline was observed in dementia-related domains of everyday skills, memory and orientation, and personality and behaviour while adjusting for the effect of age, sex, ID level, *APOE* genotype, and CVD. 

## 4. Discussion

People with DS are at an ultra-high risk of developing AD as they grow older. At present, there are no effective interventions to significantly delay or prevent the onset of this disease. As such, it is vital to identify any potential modifiable factors to try and reduce the burden of AD. In studies of SOAD, low levels of PA have been suggested as a risk factor associated with cognitive decline and dementia, although the results in the literature are mixed. Similarly, studies involving mice models and participants with Down syndrome have demonstrated that both regular exercise and acute bouts of activity can have a positive impact on cognitive function but there is limited information as to how PA may impact dementia related symptoms. 

Our study aimed to address this knowledge gap by using the CAMDEX-DS questionnaire to evaluate longitudinal associations between day-to-day levels of PA and decline in five AD related domains whilst controlling for key covariables. We found a positive association between engaging in moderate and high levels of exercise and maintenance of memory, personality, and behaviour, and everyday skills in our Down syndrome cohort.

Cross-sectionally we found that moderate levels of PA were associated with a significantly lower risk of reported decline in everyday skills. High levels of PA were associated with a reduced risk of personality and behaviour decline. Whilst the association with moderate exercise was only observed at T1, the association between high levels of PA and reduced risk of personality and behaviour decline was robust across both T1 and T2. Longitudinally, moderate levels of PA were associated with a reduced risk of decline in memory and orientation two years later. These results suggest that moderate and high levels of PA may confer protective effects against dementia-related decline and that these effects could have an enduring impact on risk. 

Based on our data, it therefore appears that regular moderate and high intensity exercise could reduce the risk of clinically detectable decline in a DS population with possible long-term benefits. This is the first study to consider the longitudinal association of regular exercise and AD-related decline in a relatively large cohort of people with Down syndrome. Our results show promise that lifestyle factors related to the risk of SOAD may also be relevant for delaying symptom onset in Down syndrome, despite the genetic driver behind AD in this population. 

These findings are consistent with the proposed neuromodulatory effects of PA. It has been suggested that engaging in regular exercise promotes a number of changes in the brain. For example, exercise has been positively associated with hippocampal volume, an area that is implicated in learning and memory decline in AD [52,53,54]. Other proposed mechanisms include alterations in cerebral blood flow [55,56,57], increasing neurotrophic factors such as BDNF in the brain [58,59], protecting neural tissue and promoting neurogenesis [60], strengthening neural connections and networks, and reducing inflammation and related tissue degeneration [61]. It has also been suggested that cardiovascular risks may be related to the progression to AD and increased amyloidosis in brain tissue, although the picture remains unclear [62,63]. We could not examine these factors in the present study due to a lack of neuroimaging data. Consideration also needs to be given to potential psychological, social, and environmental factors associated with PA, which may modulate some of the positive cognitive impact and warrant further study [64,65].

In our study, we found no association between the presence of cardiovascular disease with decline. This may be due to the specific cardiovascular profile in Down syndrome where individuals often have lower blood pressure and less atherosclerotic disease when compared to the general population [27]. It may also suggest that the associations between PA and risk of cognitive decline are not mediated simply by reducing cardiovascular risk factors. This highlights the potential for PA to exert its effects directly by modulating pathways in the brain. It also demonstrates the benefits of working with people with Down syndrome when studying AD. They represent a specific model of AD that could help disentangle the indirect impact of exercise on cardiovascular disease from direct neuroprotective effects.

Our study has a number of strengths. Cognitive assessments used in other studies have often shown only small magnitude changes with floor effects making it difficult to accurately assess response following an intervention. In our design we chose to use the CAMDEX-DS questionnaire which can detect longitudinal changes over a period of years, using the individual’s own best ever level of functioning as a baseline. Our sample size was relatively large, and included people with all levels of ID. Our information about PA is naturalistic and representative of what individuals perform on a day-to-day basis, giving our results real-world relevance. This means that the “high intensity” level of PA we studied is achievable by people with DS in the community. Exercise studies often employ highly structured and specific routines that are not feasible for individuals to incorporate into their daily lives.

We acknowledge certain limitations. The exercise data was taken from semi-structured interviews and not collected using a standardised tool. On one hand, this prevented us from being able to analyse whether certain types of exercise may confer more or less benefit to individuals. On the other hand, it means that our data is a realistic representation of the level of PA that adults with DS actually engage in. We also used informant data rather than direct cognitive assessment. This was a deliberate choice to avoid possible floor effects and allow for the inclusion of a representative sample of people with Down syndrome, including those with severe/profound ID who often score at floor or are unable to engage in direct assessment. Finally, it is difficult to ascertain the impact of social contact and other environmental factors during PA, particularly when exercise is done in a group environment. It is possible that this social aspect of PA could be contributing to the observed effects.

We excluded participants with a diagnosis of dementia. Primarily this was to allow focus on the effects of PA on early cognitive changes prior to diagnosis. This approach also helped to avoid confounding effects. People with dementia generally engage in less PA due to the physical and cognitive changes and increased frailty associated with the disease [66,67]. It would therefore be difficult to interpret the associations between levels of PA and cognitive decline in people with dementia. However, given the potential neuroprotective effects of PA on maintaining cognition it may be that are our results are also applicable to those already diagnosed with AD and could help maintain their daily functioning and quality of life for longer. Further work is needed in this area.

Our findings have significant implications for the Down syndrome community and highlight the importance of promoting healthy lifestyle and exercise alongside the search for pharmacological treatments of AD. People with Down syndrome can engage in less PA than their peers, and in our sample only 14% engaged in high levels of exercise, reducing to 9% at T2. There are concerns that barriers remain, which prevent people with Down syndrome engaging in exercise, including stigma, lack of reasonable adjustments, assumptions about the physical capabilities of people with Down syndrome, and a lack of opportunities and access [68,69]. It is vital that people with Down syndrome are supported to engage in regular exercise, and that structures are in place to encourage and support this. It can be tempting to try and find the “optimum” type, timing, and duration of exercise to provide the most benefit for individuals, but our results suggest that any substantial PA of moderate or high intensity may be of benefit. Our priority should therefore be on supporting people with Down syndrome to be healthy and active in whichever way they enjoy.

Future work should focus on the effect of PA on direct measures of cognition, the associations with neuropathological changes using blood and imaging biomarkers, and the effect of exercise at different life stages. In addition, more in-depth analysis of the type and duration of PA would help draw conclusions as to the potential role that social contact and other factors may play in maintaining cognition, and whether certain types of activity provide greater cognitive protection than others. In this exploratory work we examined the association between longitudinal change in cognition and exercise within the LonDowns cognitive test battery [45]. Due to a limited sample size and missing data, our analyses were underpowered; however, there was preliminary evidence to suggest cognition may be affected by exercise in Down syndrome. While the overall model was not significant, participants who engaged in moderate intensity exercise showed less change in their raw KBIT-2 verbal scores (*p* = 0.046) between the two time-points compared to those who engaged in low levels of exercise, adjusting for the effect of age, sex, and ID level and APOE status.

We have presented preliminary data regarding the potential positive impact of PA on AD-related cognitive decline, which is achievable for people with Down syndrome within everyday routines. Clinical trials in this area would therefore be justified to determine if engaging in exercise can lead to realistic improvements in maintaining functioning and delaying dementia onset in Down syndrome and to help develop consensus guidance in this area.

## Figures and Tables

**Table 1:** Demographics of participants at T1 and T2.

		Frequencies (%)	
		T1	T2
*n*		214	91
Follow-up time in months (mean, S.D)			24.12 (0.81)
Age (mean, S.D)		46.11 (10.47)	49.04 (6.32)
Gender	Female	92 (43.0)	37 (40.7)
	Male	122 (57.0)	54 (59.3)
Ethnicity	Bangladeshi	1 (0.5)	0 (0.0)
	Black African	4 (1.9)	2 (2.2)
	Black Caribbean	7 (3.3)	5 (5.5)
	Black other	1 (0.5)	1 (1.1)
	Chinese	2 (0.9)	1 (1.1)
	Indian	3 (1.4)	1 (1.1)
	Mixed black African/white	1 (0.5)	1 (1.1)
	Mixed other	1 (0.5)	0 (0.0)
	Other/not available	2 (0.9)	2 (2.2)
	White British	185 (86.4)	74 (81.3)
	White Irish	3 (1.4)	2 (2.2)
	White other	4 (1.9)	2 (2.2)
ID level	Mild	75 (35.7)	33 (36.3)
	Moderate	96 (45.7)	37 (40.7)
	Severe/profound	39 (18.6)	21 (23.1)
*APOE* genotype	Non-ε4 carriers	149 (77.2)	68 (80.0)
	ε4 carriers	44 (22.8)	17 (20.0)
CVD	No	147 (72.4)	59 (64.8)
	Yes	56 (27.6)	32 (35.2)
High cholesterol	No	203 (98.1)	89 (97.8)
	Yes	4 (1.9)	2 (2.2)
Smoking	Ex-smoker	3 (1.4)	2 (2.3)
	No	209 (98.1)	84 (96.6)
	Yes	1 (0.5)	1 (1.1)
Alcohol	Any alcohol consumption	68 (31.9)	31 (34.8)
	No alcohol consumption	145 (68.1)	58 (65.2)
Exercise levels	Low intensity	78 (37.7)	31 (36.0)
	Moderate intensity	100 (48.3)	47 (54.7)
	High intensity	29 (14.0)	8 (9.3)

**Table 2:** Changes in CAMDEX-DS domain at T1 and T2.

CAMDEX-DS Domain		T1	T2
*n*		214	91
Everyday skills	No	147 (70.3)	62 (68.1)
	Yes	62 (29.7)	29 (31.9)
Memory and orientation	No	132 (62.6)	63 (69.2)
	Yes	79 (37.4)	28 (30.8)
Other cognitive skills	No	132 (63.5)	57 (62.6)
	Yes	76 (36.5)	34 (37.4)
Personality and behaviour	No	129 (61.4)	51 (56.0)
	Yes	81 (38.6)	40 (44.0)
Self-care	No	152 (73.8)	66 (72.5)
	Yes	54 (26.2)	25 (27.5)

## Data Availability

The datasets used and/or analysed during the current study are available from the corresponding author on reasonable request.
